# Cosmetic outcomes of surgical neck humeral osteotomy for the flail limb: a case report

**DOI:** 10.1016/j.xrrt.2026.100776

**Published:** 2026-05-15

**Authors:** Davis Howell, Srinath Kamineni

**Affiliations:** a Department of Orthopaedic Surgery and Sports Medicine, University of Kentucky College of Medicine, Lexington, KY, USA; b Department of Orthopaedic Surgery, Indiana University School of Medicine, Evansville, IN, USA; c Wabash General Hospital Orthopaedics & Sports Medicine, Mount Carmel, IL, USA

**Keywords:** Upper extremity, Amputation, Surgical neck, Flail limb, Transhumeral, Cosmetic

## Introduction

**Figure 1:**
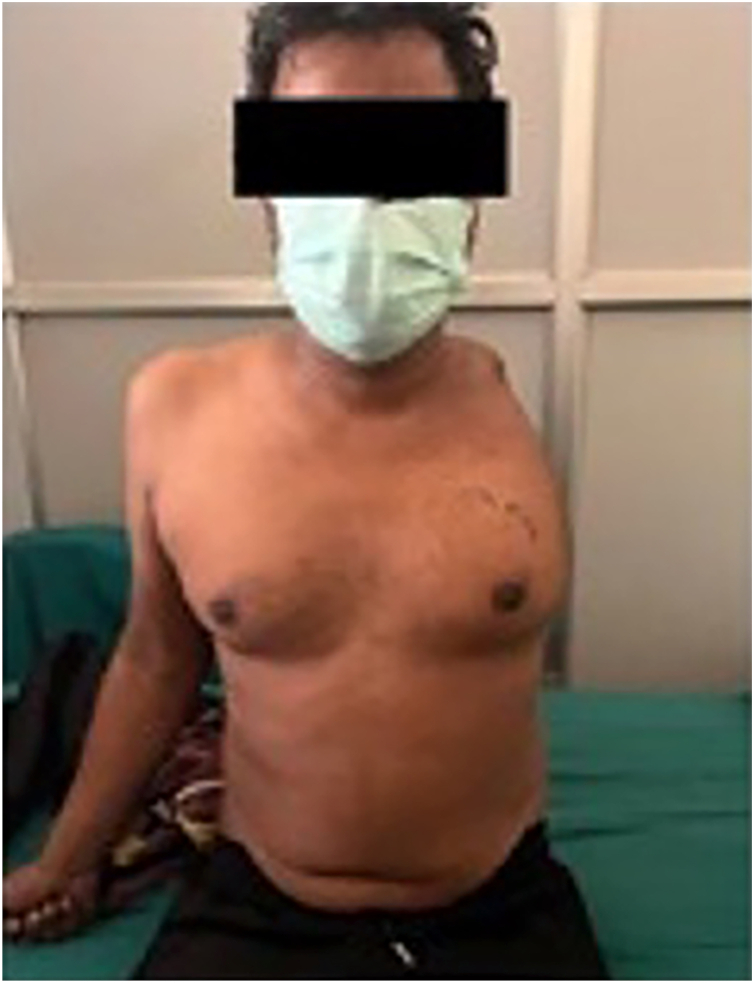
Patient who underwent forequarter amputation following traumatic scapulothoracic dissociation. Reprinted with permission from Upreti and colleagues.^13^

**Figure 2:**
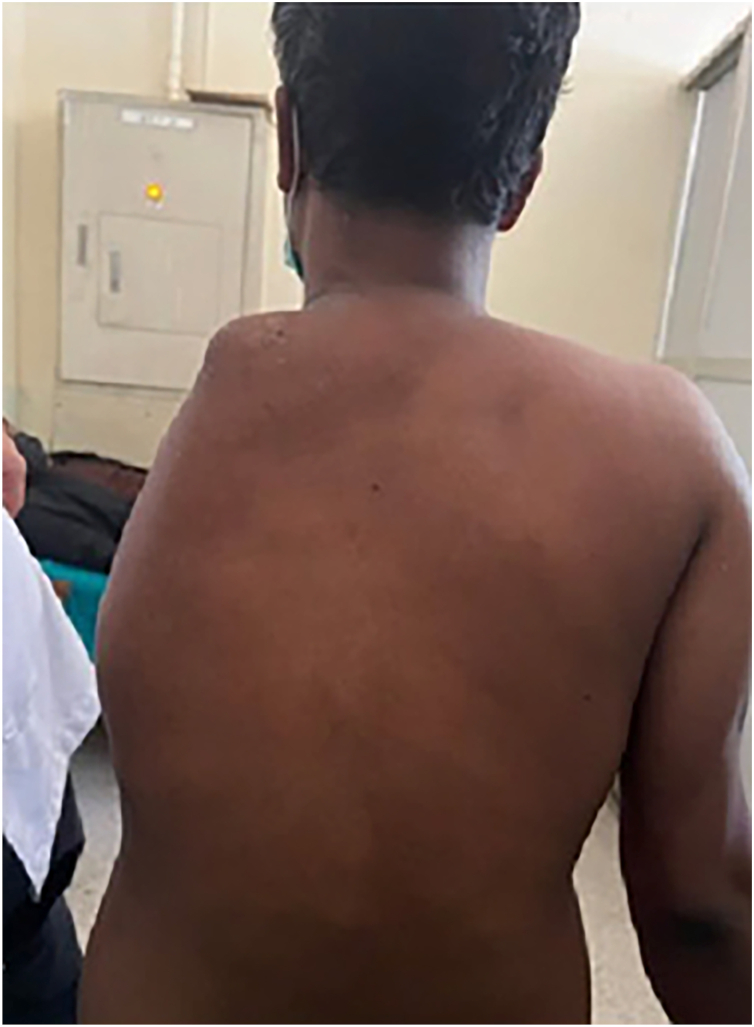
Patient who underwent forequarter amputation following traumatic scapulothoracic dissociation. Reprinted with permission from Upreti and colleagues.^13^

Upper extremity amputations are performed for multiple indications, ranging from malignancy of the arm or shoulder girdle to traumatic injuries that severely limit function.^3^^,^^6^^,^^11^^,^^14^ When considering an amputation, the level at which the procedure is performed must be carefully considered and discussed with the patient. If function of the limb has been totally compromised due to trauma or neurological injury, it may be necessary to remove the entire extremity to alleviate the added weight on the torso. One way this has traditionally been achieved is via the interscapulothoracic, or forequarter, amputation, where the entire arm is removed along with the scapula^3^^,^^6^^,^^13^^,^^14^ (Figures 1 and 2, reprinted with permission from Upreti and colleagues^13^). Although this technique accomplishes its purpose, it has the disadvantage of disfiguring the trunk of the patient and leaving an asymmetric hemithorax. This presents additional challenges to patients when adjusting to life after surgery, notably the psychological distress of the physical asymmetry and all previous clothing no longer fitting correctly. As a result of this shortcoming, several techniques have been developed to recreate the contour of the shoulder in full upper extremity amputations and thus improve cosmesis, making use of fillet flaps and even osteomyocutaneous flaps using the elbow joint.^1^^,^^5^^,^^10^^,^^12^

This case report introduces an uncommon technique for amputation at the level of the proximal humerus, in which the osteotomy is performed through the surgical neck of the humerus. Because the glenohumeral joint is kept intact, this technique preserves an anatomic contour of the shoulder and thoracic width, while removing the weight of a nonfunctional extremity causing significant discomfort. While techniques similar to this one have been described in previous literature, there are currently no records of an amputation at the level of the proximal humerus solely for the indication of neurological injury and a nonfunctional limb.^3^ Rather, glenohumeral arthrodesis, above-elbow amputation, or a combination of the 2 are common strategies for management of upper extremities with severe neurological damage such as brachial plexus palsy.^1^^,^^2^^,^^7^, ^8^, ^9^ These techniques allow for prosthetic use with the residual limb, and arthrodesis aims to counteract glenohumeral subluxation caused by the weight of the flail limb.

## Case description

**Figure 3:**
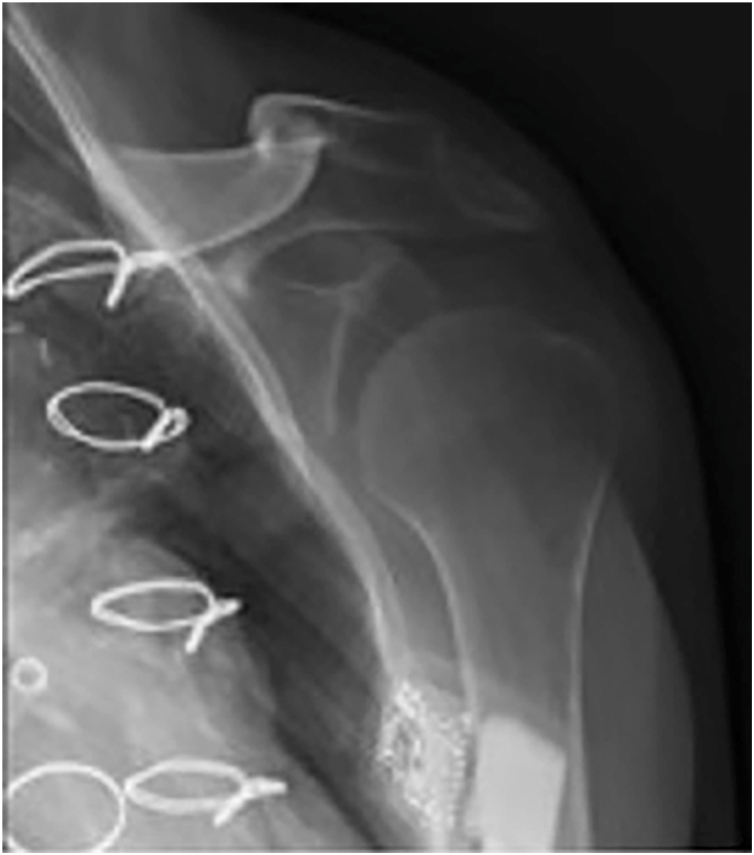
Pre-operative radiograph showing inferiorly subluxed humeral head.

A 34-year-old man presented to the clinic with severely limited function in his left upper extremity. The patient had an extensive cardiac history and suffered a middle cerebral artery stroke status-post revision aortic root reconstruction to repair pseudoaneurysm of the ascending aorta 2 years before presentation. As a result, the function of his left arm was significantly diminished, unusable for daily activities. The extremity was subtotally paralytic, as the patient was able to abduct approximately 30 degrees at the shoulder with minimal scapular control. The patient's main complaint was a painful “dragging” sensation created by the weight of the limb on his trunk (Fig. 3). Magnetic resonance imaging without intravenous contrast was obtained to assess the rotator cuff and glenohumeral joint. This scan showed extensive fatty infiltration of the shoulder musculature with borderline to mild supraspinatus atrophy, irregularity, and blunting of the anterior and posterior glenoid labrum, and mild to moderate glenohumeral osteoarthrosis. Multiple sites were discussed for the procedure, including forequarter, above, and below-elbow amputation options. Glenohumeral arthrodesis was proposed, but he believed that this procedure would not address the weight of his limb. The amputation level was secondarily discussed with regard to cosmesis and postsurgical clothing, and a proposal of a humeral surgical neck-level amputation was mutually agreed upon.

The procedure consisted of beach chair positioning and fish-mouth incision distal to the surgical neck of the left humerus. The biceps brachii was identified, and the musculocutaneous nerve was suture ligated and transected, along with the brachial artery, median, and ulnar nerves. The deltoid insertion was detached from the humerus, and the deltoid was preserved for lateral flap closure. Medial dissection was then continued into the posterior compartment of the arm, at the distal border of the subscapularis, where the radial nerve was ligated and transected. The dissection was continued circumferentially through the brachialis and triceps, with radiocautery, at the surgical neck level. The sharp bony edges were chamfered to avoid soft tissue irritation. The deltoid muscle belly was wrapped over the residual bone and secured to the pectoralis major muscle belly anteriorly and latissimus and teres major posteriorly with silk suture to create a closed and contoured muscle sleeve and counter inferior subluxation of the humeral head. Finally, the skin flaps were reapproximated, without a drain, with a combination of Vicryl and nylon closure.

**Figure 4:**
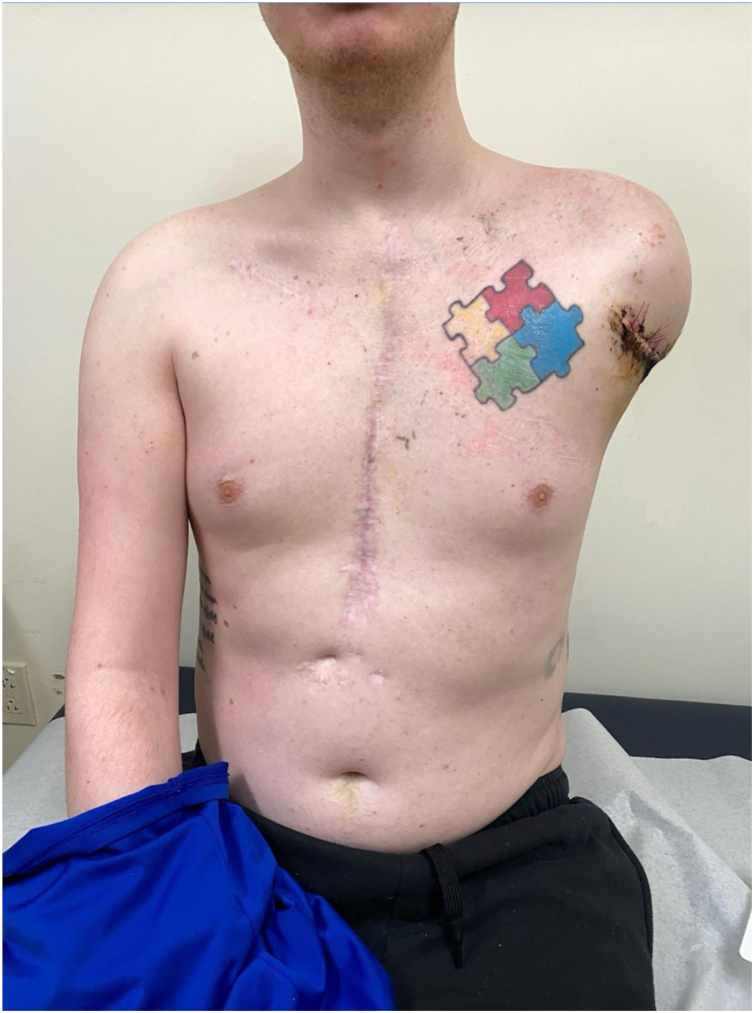
Patient at 2-week post-operative visit demonstrating symmetric hemithorax following amputation.

**Figure 5:**
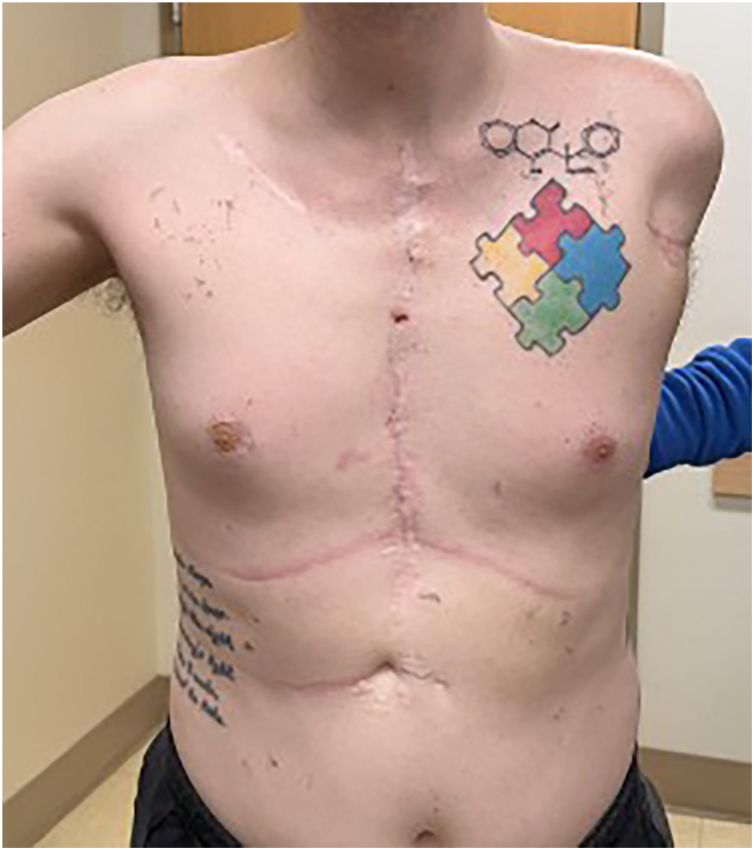
Patient 7 months post-operation with healed wound.

At the 2-week postoperation clinic visit, the patient reported significant improvement in symptom burden as a result of the procedure, although experiencing phantom pain from the amputation site (Fig. 4). The patient was lost to follow-up for several months post-op, due to multiple cardiac and trauma-related events. At an 8-month visit, he rated his baseline pain level a 0 out of 10 but did report occasional but significantly decreasing frequency of phantom pains. His primary pre-operative complaint of a “dragging” sensation created by the weight of his limb on the scapula had completely resolved and was happy with his “shape” (Figure 5, Figure 7, Figure 8). Overall, he was extremely pleased with the results of the procedure and has not had to purchase new clothing due to improper fit.

**Figure 6:**
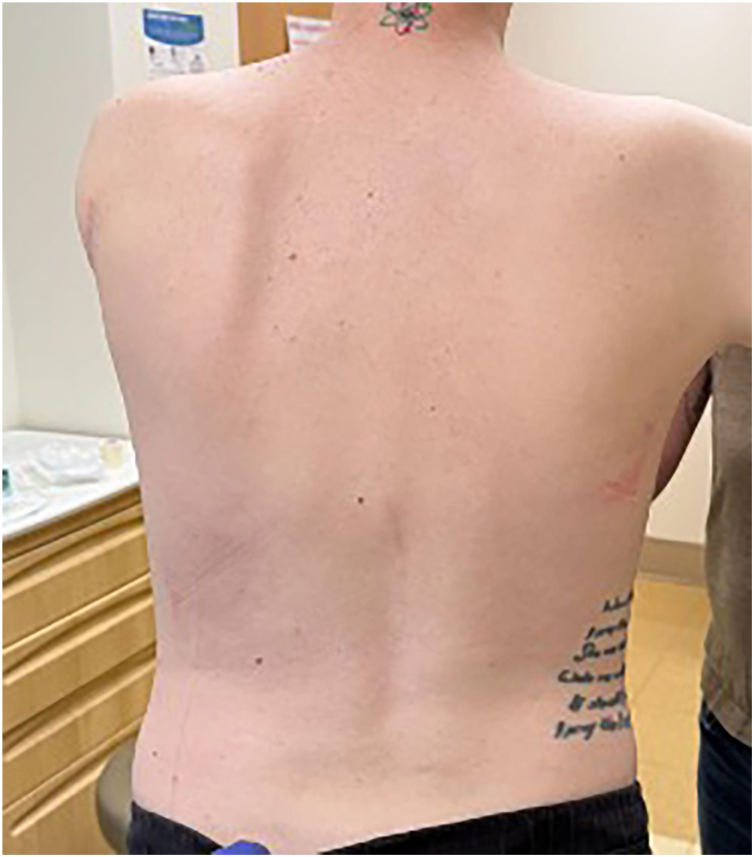
Patient 7 months post-operation with healed wound.

**Figure 7:**
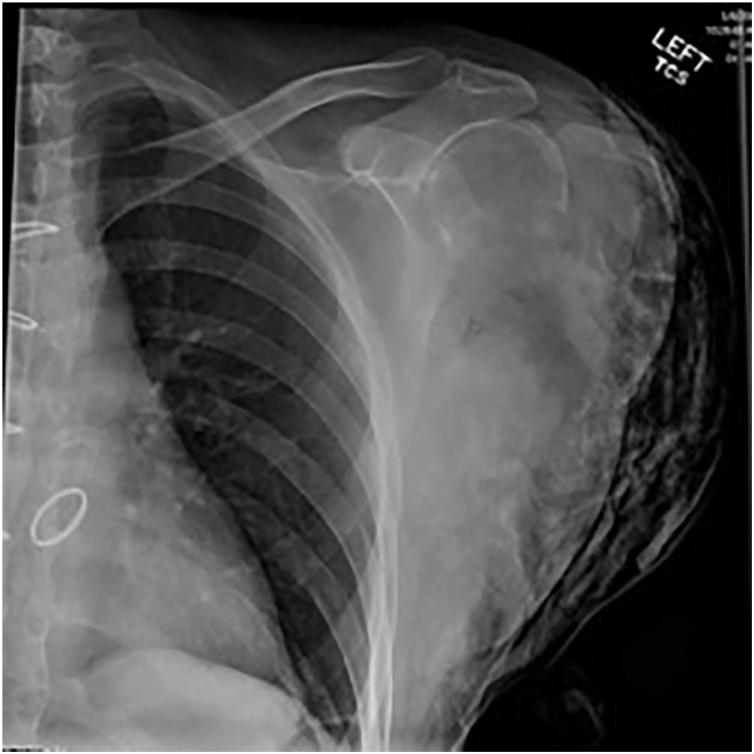
Post-operative anterior-posterior radiograph of the left shoulder.

**Figure 8:**
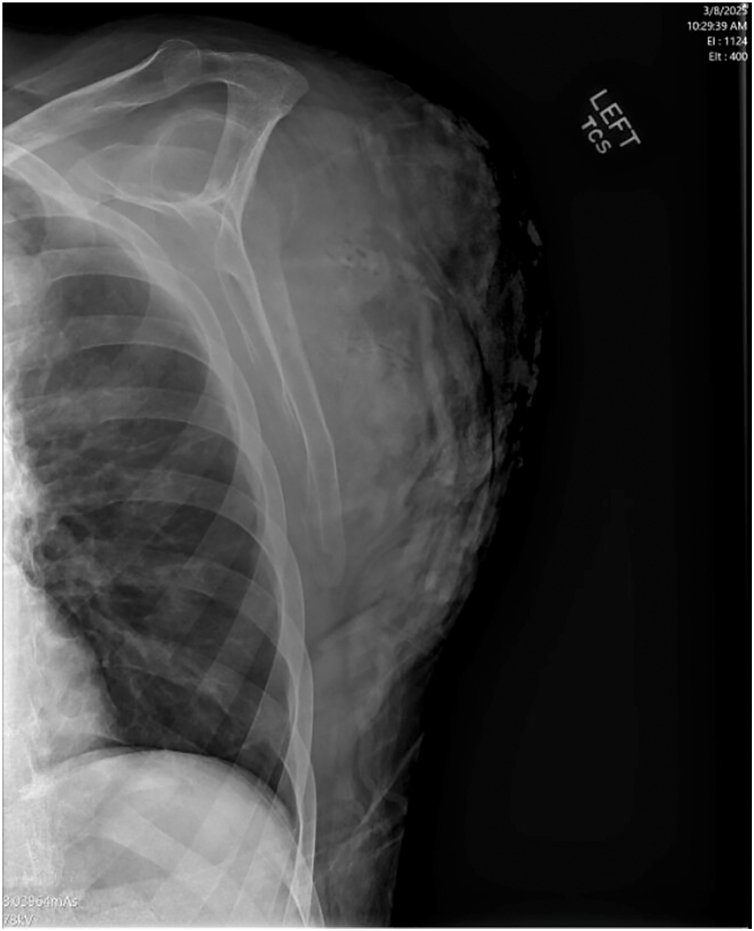
Post-operative scapular Y radiograph of the left shoulder.

## Discussion

In cases where upper extremity function is compromised and amputation is considered, the question of whether to keep a portion of the limb or remove it entirely must be addressed. While forequarter amputation (FQA) addresses removing the excess burden of the arm, one of the primary complaints from the patient perspective is the resulting asymmetry of the hemithorax due to the entire scapula and humerus being removed. This not only leaves patients with an unnecessarily disfigured appearance (Figures 1 and 2, reprinted with permission from Upreti and colleagues^13^) but also poses great difficulty with fitting into normal clothing. Overall, this adds to the psychological burden and financial burden of buying new clothes for a patient who is already undergoing a significant event. Conversely, the novel technique presented in this report spares the glenohumeral joint and maintains the original contour of the shoulder via the remaining humerus proximal to the surgical neck (Figs. 5 and 6). This helps patients maintain as much normality as possible following surgery and avoid the financial strain of needing to purchase new clothing or seek out prostheses to accommodate an asymmetric hemithorax.

The argument could be made that an above- or below-elbow-level amputation would serve the same purpose as one at the surgical neck while also avoiding an asymmetric hemithorax. These procedures have been shown to be useful in cases of bone or soft tissue malignancy of the upper extremity with intact function and have also been indicated to improve pain in patients with severe brachial plexus injuries.^8^^,^^11^ However, amputations that retain more than minimal humeral length are not always ideal when arm function is lost, as they leave a portion of a completely flail limb that is only causing discomfort to the patient. Rather, removing the excess weight of the entire arm is more likely to provide patients with maximum relief. Performing the transhumeral amputation through the surgical neck of the humerus provides patients with the best of both worlds: alleviating extensive pain for the patient while also maintaining the symmetry of the torso and easing the post-operative transition to daily life.

While this procedure led to a satisfying result for this patient, both neurological functional status and goals of function must be carefully examined when considering amputation at the humeral surgical neck level. This patient's shoulder and scapular musculature were not entirely paralytic, maintaining minimal scapular control and arm abduction. This preserved innervation allowed for a more pleasing cosmetic result rather than the potential for a residual winged scapula if his upper extremity was completely paralyzed. Additionally, the small segment of remaining humerus after this procedure would make prosthesis use quite difficult. However, this patient's primary goal was reduction of limb weight rather than maintaining arm functionality. Long-term functionality goals must be clearly established with patients to determine if a more proximal amputation is a viable option.

When performing a transhumeral amputation in a patient with lack of dynamic shoulder stability, residual post-operative glenohumeral subluxation and degenerative change are theoretical concerns. However, the current literature appears to support improvement in pain from subluxation following transhumeral amputation, indicating that the glenohumeral instability is driven primarily by the weight of the limb, rather than lack of dynamic stability.^1^^,^^4^ There is scarce literature detailing the incidence of glenohumeral osteoarthritis following transhumeral amputation, particularly for the indication of a flail upper extremity. This could be due to reduced compressive forces on the glenohumeral joint from paralyzed rotator cuff muscle and the absence of distal load-bearing following amputation.

## Conclusion

Upper extremity amputation at the level of the humeral surgical neck can provide benefits for patients with severely limited limb function who wish to find relief from the weight of a nonfunctional extremity. Due to preserved native shoulder contour, this procedure also provides an improved cosmetic outcome and easier transition post-operatively in comparison to more radical techniques like FQA.

## Disclaimers:

Funding: No funding was disclosed by the authors.

Conflicts of interest: The authors, their immediate families, and any research foundations with which they are affiliated have not received any financial payments or other benefits from any commercial entity related to the subject of this article.

Patient consent: Obtained.

## Declaration of AI and AI-assisted technologies in the writing process

During the preparation of this work, the author(s) used ChatGPT to assist with literature review. All relevant articles were personally reviewed by the author(s). After using this tool/service, the author(s) reviewed and edited the content as needed and take(s) full responsibility for the content of the published article.
